# Association between carriers of the G allele of the + 45T> G variant of the ADIPOQ gene (*rs 2241766*) and the cardiometabolic profile in sickle cell trait

**DOI:** 10.1016/j.heliyon.2021.e06443

**Published:** 2021-03-11

**Authors:** Jamila Benvegnú Bruno, Emanuelle Schneider Dal Ponte, Vanessa Retamoso, Patrícia Maurer, Lyana Feijoó Berro, Vanusa Manfredini, Jacqueline da Costa Escobar Piccoli

**Affiliations:** a Postgraduate Program in Biochemistry, Federal University of Pampa, BR 472, Km 592, P.O. BOX 118, Zip Code 97508-000, Uruguaiana, Rio Grande do Sul, Brazil; b Postgraduate Program in Pharmaceutical Sciences, BR 472, Km 592, P.O. BOX 118, Zip Code 97508-000, Uruguaiana, Rio Grande do Sul, Brazil

**Keywords:** Adiponectin, Metabolic syndrome, Sickle cell trait, rs 2241766, +45T>G variant in ADIPOQ gene

## Abstract

**Aims:**

investigate the association between the +45T > G variant of the ADIPOQ gene and the metabolic syndrome (MS) in patients with sickle cell trait (SCT). 33 patients with SCT and 35 control group participated in the study. Lower levels of HDL and adiponectin were observed in patients with G allele and sickle cell trait. There were no differences between the prevalence of MS between the groups and there was no association between the +45T > G variant of the ADIPOQ gene and MS risk allele.

**Materials and methods:**

Participants with and without sickle cell anemia answered a questionnaire, performed anthropometric and laboratory analyzes. They were genotyped for the +45T > G variant of the ADIPOQ gene and evaluated for the presence or absence of metabolic syndrome. The study was approved by the Research Ethics Committee of UNIPAMPA (RS/Brazil).

**Key findings:**

The GG + TG genetic model, it was associated with lower levels of adiponectin and HDL cholesterol in the SCT group. There was no association between the other studied markers and MS.

**Significance:**

For the first time, an association was demonstrated between the G allele of the +45T > G variant of the ADIPOQ gene and a worse cardiometabolic profile (lower serum concentrations of adiponectin and HDL cholesterol) in patients with sickle cell trait.

## 1 Introduction

Hemoglobinopathies are autosomal recessive pathologies associated with hemoglobin protein synthesis [1,2] and sickle cell anemia (SCA) is one of them [3]. Heterozygosity for the hemoglobin S (HbS) gene is the carrier of the sickle cell trait (SCT), which presents a relatively common and clinically benign condition, inheriting the hemoglobin A (HbA) gene and the HbS gene [4]. In Brazil, as in the world, the sickle cell trait (SCT) is the most common variant of hemoglobin, with a general prevalence of 2.49% of Brazilians, but among African ascendance or mestizo ascendance, which correspond to more than half of the population in Brazil, this prevalence can reach 4.1% and 3.6%, respectively [5,6]. In addition to the genetic conditions of individuals, it is known that, in certain situations, the severity of some types of diseases can be affected in this group of patients. In this context, metabolic syndrome (MS) can be considered. MS includes multiple factors such as abdominal obesity, insulin resistance, hypertension and dyslipidemia [7]. The genesis of most clinical manifestations of MS can be increased in patients with HbS, which is especially linked to interrelated mechanisms, such as erythrocyte adhesion, granulocytes, monocytes and platelets to the endothelium. In addition, chronic inflammatory phenomena and cytokine production may also be correlated [8,9,10]. Furthermore, adipose tissue also has the secretory ability of various bioactive molecules including adiponectin which is a hormone that has antiatherogenic and anti-inflammatory activity and can be considered as an important biomarker [11]. In humans, adiponectin is synthesized by the AdipoQ gene, on chromosome 3 (3q27) and has polymorphisms such as the single nucleotide polymorphism (SNP) + 45T > G (rs 2241766), located in exon 2, whose variations may be related to serum changes in adiponectin [12], obesity [13,14] and insulin resistance [15]; however, its role in SCT patients with MS has not yet been studied. Considering two potentially pro-inflammatory conditions of the vascular endothelium (presence of HbS and MS), the present study aims to investigate whether there is an association between the +45T > G variant in ADIPOQ gene and the metabolic syndrome in patients with SCT to elucidate possible inflammatory mechanisms related to the cardiometabolic health of these patients.

## 2 Material and methods

### 2.1 Research participants

Blood donors, from the Blood Bank of the city of Uruguaiana/RS/Brazil, were invited to participate in the study by telephone, from August to December 2016. During this period, 68 donors agreed to participate in the study and were divided into two groups, patients with sickle cell trait (n = 33) and control group without sickle cell trait (n = 35). All participants underwent an interview, anthropometric assessment, blood pressure measurement and fasting blood collection for laboratory analysis. Individuals diagnosed with other hemoglobinopathies were excluded from the study.

### 2.2 Ethic

This study was approved by the Research Ethics Committee of Universidade Federal do Pampa (Federal University of Pampa), protocol number 977,827, and has been carried out in accordance with the Code of Ethics of the World Medical Association of Helsinki). All participants agreed to voluntarily participate in the study and signed an Informed Consent Form and had their privacy rights guaranteed.

### 2.3 Anthropometric assessments

Weight (kg) was quantified by means of a digital scale (Omron HBF - 514), height (cm) by means of a portable stadiometer (Balmak) and abdominal circumference (cm) and hip circumference (cm) by measuring tape.

### 2.4 Criteria for evaluation of metabolic syndrome (MS)

MS was estimated using the criteria established by NCEP-ATP III [16] that advocates the diagnosis of metabolic syndrome in the presence of at least three of the five criteria: waist circumference ≥88cm for women, or ≥102cm for men; HDLc<50 mmol/dL for women, or <40 mmol/dL for men; triglycerides ≥150 mmol/dL; blood pressure with cut-off values considering 130/85mmHg; and fasting glycemia ≥110 mmol/dL.

### 2.5 Laboratory analysis

#### 2.5.1 Biochemical parameters

Peripheral blood samples were collected in a 12-hour fasting. These were centrifuged for 15 min at 3000g. Aliquots of serum and plasma were separated for the following biochemical analyzes: levels of total cholesterol, HDL, LDL, Triglycerides and glucose were measured with kits from the Labtest Company on automated equipment ChemWell T Labtest (Lagoa Santa/MG, Brazil).

#### 2.5.2 Hematologic parameters

Complete blood counts and platelet counts were performed using the Sysmex®KX 21N automatic counter. The separation of fractions of hemoglobin and the confirmation of the presence or absence of the sicle trait was performed in the equipment of high performance liquid chromatography (HPLC) Bio-Rad D10.

#### 2.5.3 Analysis of adiponectin

Adiponectin levels were measured by Enzyme-Linked Immunosorbent Assay (ELISA), using commercial kits (R&D Systems, Inc., Minneapolis, MN, USA), according O'Rourke *et al.* [17].

#### 2.5.4 Detection of SNP +45T > G

The genomic DNA was isolated from peripheral blood leukocytes using the Wizard® Genomic DNA Purification Kit - Promega. The +45T > G variant in ADIPOQ gene (*rs2241766*) was determined through Polymerase Chain Reaction and Restriction Fragment Lenght Polymorphism (PCR-RFLP). The Polymerase Chain Reaction (PCR) used the primers: (F) 5′-GCAGCTCCTAGAAGTAGACTCTGCTG-3′ and (R) 5′-GCAGGTCTGTGATGAAAGAGGCC-3′ as described in El-Shal, *et al.* [12] After amplification, an electrophoretic run was performed to confirm the size of the amplified fragment (372pb). For genotyping, the restriction enzyme SmaI (Invitrogen, Carlsbad, California). The digested fragments were analyzed on agarose gel, 2.5%. The gel was visualized in photodocumentator Gel Doc XR + System BIORAD being observed the fragments of the following size: allele T = 372pb, allele G = 219pb, and 153pb [18].

### 2.6 Statistical analysis

The data were tabulated and analyzed by the statistical program SPSS, version 20.0. The Hardy-Weinberg equilibrium test was performed using ARLEQUIN software (Geneva, Switzerland).The quantitative variables were analyzed by mean/standard deviation and the differences between the means evaluated by Student's t-test. The samples were tested for normality by the Kolmogorov-Smirnov test (p > 0.05 considered normal distribution) and homogeneity was tested by Levene's test (p > 0.05 considered homogeneous). All variables met criteria for normality and/or homogeneity. Qualitative variables were made by frequency analysis (%), followed by chi-square test. The power of the sample test was calculated using the "pwr" package of software R, version 3.6.1 (The R-project for statistical computing). The significance level of 5% was used and the result obtained was a power of 90.12%.Were considered significant associations with p < 0.05.

## 3 Results and discussion

The present study demonstrated, for the first time, the sickle cell trait associated with a multifactorial disease, such as MS, and a possible relationship with the SNP of the adiponectin synthesizing gene, being a new debate on issues related to the cardiometabolic health of this group. The study included 68 individuals, 33 (48.5%) with SCT and 35 (51.5%) without SCT (control group) and the general data for the groups are shown in Table 1. There were no statistical differences between groups regarding sex, age and frequency of MS. The observed differences were in relation to hematological and biochemical data. The SCT group had lower levels of hematocrit, MCV and WBC than the control group. In addition, the SCT group showed significantly greater changes in MCHC and platelet count than in the control group. It is important to note that SCT has a benign condition, usually asymptomatic, without physical abnormalities and with life expectancy equal to that of the normal population. As we can see in this study, laboratory tests do not find findings altered to the disease; showing that the group does not have anemia and with results within the reference values. Previous studies report that red blood cell survival is normal, without hemolysis or other laboratory abnormalities [19]. Serjeant [20] demonstrated that hematological findings are normal in SCT, with hemoglobin levels ranging from 13 to 15 g/dL and MCV from 80 to 90 fL, which corroborates what was found in the present study, which presented hemoglobin levels 13.3 (±1.3) and VCM 83.2 (±3.6) for the group with SCT. Summer et al. [21] found similar results between hematological analyzes performed on African immigrants in the Americas asymptomatic, with the hemoglobin and hematocrit concentrations of the groups within normal values.

**Table 1:** Clinical and laboratory characteristics of patients with sickle cell trait and control group.

Characteristics	Sickle cell trait N = 33	Control Group N = 35	*P*
*Quantitative variables*			
Age (year)	35.5 (±12.7)	37.3 (±11.8)	0.57
Body mass index (Kg/m^2^)	27.2 (±4.7)	29.06 (±5.7)	0.16
Waist circumference (cm)	94.4 (±12.4)	95.2 (±12.8)	0.81
Systolic blood pressure (mm Hg)	126.6 (±12.0)	135.4 (±26.5)	0.10
Diastolic blood pressure (mm Hg)	83.5 (±15.6)	86.3 (±16.5)	0.47
Total cholesterol (mmol/dL)	158.7 (±40.6)	180.5 (±180.5)	0.025
HDL-Cholesterol (mmol/dL)	47.9 (±21.0)	50.8 (±9.2)	0.45
	Men 44.1 (±16.4)	Men 47.9 (±8.4)	
	Women 51.9 (±12.0)	Women (±9.4)	
Triglycerides (mmol/dL)	121.4 (±58.3)	141.2 (±91.7)	0.29
Fasting blood glucose (mmol/dL)	86.0 (±14.0)	109.5 (±14.8)	<0.001
Hemoglobin (g/dL)	13.3 (±1.3)	13.4 (±1.2)	0.92
RBC (10^3^/mm^3^)	4.6 (±0.61)	4.6 (±0.55)	0.95
Hematocrit (%)	38.0 (±3.7)	40.4 (±3.8)	0.013
MCV (fL)	83.3 (±3.6)	87.2 (±4.7)	<0.01
HCM (pg)	29.2 (±1.8)	28.3 (±2.3)	0.075
MCHC (g/dL)	34.9 (±1.9)	32.5 (±2.2)	<0.001
WBC	6280 (±1631.0)	6725 (±1626.6)	<0.001
Platelets count (mm^3^)	338160.0 (±129045.0)	254176.5 (±51196.2)	0.02
Hemoglobin S (g/dL)	40.3 (±1.44)	0	
Adiponectin (*μ*g.ml^−1^)	10.8 (±5.6)	9.4 (±5.6)	0.35
*Qualitative variables*			
Men	17 (51.5%)	13 (37.1%)	0.22
Women	16 (48.5%)	22 (62.9%)	
Metabolic Syndrome (NCEP)	10 (30.3%)	14 (40.0%)	0.40

RBC = Red blood cells, MCV = Mean Corpuscular Volume; HCM = Hemoglobin Corpuscular Mean; MCHC = Mean Corpuscular Hemoglobin Concentration; WBC = White blood cells. Quantitative variables: media (±standard deviation), t-student test. Qualitative variables: Chi-square test or Fisher's exact test.

Patients with SCT had significantly lower total cholesterol levels than the control group. Menezes et al. [22], in a previous study, evaluated the total cholesterol concentrations in individuals with sickle cell anemia (HbSS) and found values lower than those observed in the control group. Although total cholesterol is strongly associated with cardiovascular risk, the differences observed between the groups are within tolerable parameters. The same occurred in blood glucose, where there is a statistical difference between the SCT and the controls, but still within the reference value for normality. Thus, it was considered that the groups, despite the differences in these parameters, could be considered for the purposes of the other comparisons, also considering that there were no effects on the frequency of MS among them.

The allelic and genotypic frequencies are shown in Table 2. The studied population was in Hardy-Weinberg Equilibrium (χ^2^ = 1.192; *p* = 0.838). The frequency of the GG + TG dominant genetic model was also calculated to assess the influence of the G allele on associations.

**Table 2:** Allelic and genotypic frequencies of +45T > G variant in ADIPOQ gene in patients with sickle cell trait compared with the control group.

+45T > G variant in ADIPOQ gene	Sickle cell trait N = 33	Control Group N = 35	P
**Genotypic frequency**			
TT	23 (69.7%)	26 (74.3%)	
TG	9 (27.3%)	7 (20.0%)	
GG	1 (3.0%)	2 (5.7%)	
**Allele frequency**			
T	0.83	0.86	
G	0.17	0.14	
**Dominant genetic model**			
GG + TG	10 (30.3%)	9 (25.7%)	0.673
TT	23 (69.7%)	26 (74.3%)	

Considering the low frequency of the G allele, demonstrated in other studies and observed here too, we chose to study the influence of the presence of at least one G allele, joining the TG and GG genotypes in a group for comparison with the wild TT genotype. Thus, GG + TG and TT, were evaluated between the SCT and control groups, in order to verify the possible effect of the G allele on the main clinical and laboratory parameters studied. In the SCT group, it was possible to observe that carriers of G allele (GG + TG) had lower concentrations of HDL cholesterol and adiponectin when compared to the TT genotype (Table 3). In the control group, there was no difference between the means between the GG + TG and TT groups.

**Table 3:** Carriers of the G allele +45T > G variant in ADIPOQ gene and levels of clinical and laboratory parameters of controls and SCT groups.

Clinical and laboratory Biomarkers	Sickle cell trait N = 33		Control Group N = 35	
	GG + TG (n = 10)	TT (n = 23)	GG + TG (n = 9)	TT (n = 26)
Body mass index (Kg/m^2^)	28.6 ± 5.5	26.6 ± 4.3	31.6 ± 6.3	28.2 ± 5.4
Waist circumference (cm)	95.6 ± 16.3	93.9 ± 10.6	99.1 ± 14.2	93.8 ± 12.4
Systolic blood pressure (mm Hg)	123.3 ± 16.0	126.2 ± 10.0	121.1 ± 14.4	140.0 ± 30.7
Diastolic blood pressure (mm Hg)	88.2 ± 14.9	81.4 ± 15.7	88.4 ± 10.7	86.3 ± 18.3
Total cholesterol (mmol/dL)	160.3 ± 28.8	158.1 ± 45.4	163.2 ± 32.0	185.8 ± 37.6
HDL-Cholesterol (mmol/dL)	38.3 ± 11.8^∗^	52.0 ± 22.9	52.2 ± 10.2	50.4 ± 9.0
Triglycerides (mmol/dL)	133.9 ± 77.8	115.9 ± 51.1	128.5 ± 95.4	145.1 ± 92.2
Fasting blood glucose (mmol/dL)	82.6 ± 8.0	87.4 ± 13.5	108.0 ± 14.1	110.1 ± 15.4
Hemoglobin(g/dL)	13.3 ± 1.6	13.4 ± 1.2	12.8 ± 1.3	13.6 ± 1.4
RBC (10^3^/mm^3^)	4.5 ± 0.5	4.6 ± 0.6	4.6 ± 0.5	4.6 ± 0.6
Hematocrit (%)	38.2 ± 3.4	38.0 ± 4.0	38.8 ± 3.9	41.0 ± 3.8
VCM (fL)	82.7 ± 3.8	83.6 ± 3.6	84.3 ± 3.9	88.2 ± 4.8
HCM (pg)	28.8 ± 1.8	29.4 ± 1.8	27.8 ± 1.8	28.5 ± 2.6
MCHC (g/dL)	34.6 ± 0.9	35.1 ± 0.1	33.2 ± 2.8	32.3 ± 2.0
WBC (%)	6.2 ± 1.3	6.3 ± 1.8	7.5 ± 0.9	6.5 ± 0.6
Platelet count (mm^3^)	429100 ± 118800	315425 ± 125733	256250 ± 68217	253538 ± 46421
Hemoglobin S (g/dL)	40.0 ± 1.4	40.4 ± 1.5	0	0
Adiponectin (*μ*g.ml^−1^)	7.2 ± 5.4^∗∗^	12.3 ± 5.2	9.9 ± 4.4	9.4 ± 6.0
Metabolic Syndrome n%	4 (40)	6 (26.1)	4 (44.4)	10 (38.5)

RBC = Red blood cells, MCV = Mean Corpuscular Volume; HCM = Hemoglobin Corpuscular Mean; MCHC = Mean Corpuscular Hemoglobin Concentration; WBC = White blood cells. Quantitative variables: t-student test. Data expressed as mean ± standard deviation and analyzed by test t-student. The comparison between the frequencies of metabolic syndrome were calculated using Pearson's chi-square test. ^∗^p = 0.03 between GG + TG and TT genotype in the SCT group. ^∗∗^p = 0.02 between GG + TG and TT genotype in the SCT group.

In addition, in order to observe the influence of the G allele on MS, a comparison was made between GG + TG and TT in the SCT and in the controls. There was no statistical difference between the groups (Table 3).

Both HDL cholesterol and adiponectin can have their levels directly related to protection against cardiovascular risk, and their low concentrations are considered a cardiometabolic risk factor. In this study, it can be seen that, in the SCT group, the G allele was related to lower concentrations of HDL and adiponectin, the same did not occur in the control group. There are no previous studies associating the effect of the G allele of +45T > G variant in ADIPOQ gene on adiponectin concentrations in SCT, however, it has been shown that it can alter adiponectin concentrations in other diseases and populations. The +45T > G variant in ADIPOQ gene has been linked to the development of DM2, insulin resistance, obesity, coronary artery disease [23] and hypoadiponectinemia [24]. Paradoxically, in another type of hemoglobinopathy, beta-thalassemia, studies show that adiponectin levels are high, raising questions about their pro or anti-inflammatory role in this disease [25,26]. Despite these divergences, studies with the +45T > G variant in ADIPOQ gene have not yet been found in other hemoglobinopathies. Thus, we emphasize the importance of further studies on this topic, given the importance of vascular and inflammatory changes, possibly resulting from these changes.

Hara et al [27] reported that individuals with the TG or GG genotype at position 45 have a higher risk of developing DM2 when compared to those of the TT genotype. Therefore, individuals with the G allele of the +45T > G variant in ADIPOQ gene have lower plasma levels of adiponectin [24], which was observed in the studied SCT group. Although, without difference in the frequency of MS, the TT + TG SCT group may present a higher risk of worsening in the long-term cardiometabolic profile, and even of MS. On the other hand, Sanchés et al. [28] suggested that the presence of the G allele has a protective effect in individuals with DM2 and MS. In a previous study, Retamoso et al. [29] demonstrated that the +45T > G variant in ADIPOQ gene did not affect the frequency of MS in a group of elderly people in RS, however, no previous study with the elderly and SCT was found.

The main limitation of our study was the short period of data collection, which led to a small sample size so that our objectives could be fully clarified and, therefore, we suggest that subsequent complementary studies will be necessary to better elucidate the role of the G allele in the possible increase cardiovascular risk associated with reduced serum adiponectin and HDL levels in patients. Even so, we believe that the results demonstrated here open perspectives for new studies, which may better elucidate the role related to the risk of the G allele of the +45T > G variant in ADIPOQ gene in the cardiometabolic risk of patients with sickle cell trait.

## 4 Conclusion

There was an association between the carrier G allele of +45T > G variant in ADIPOQ gene and lower serum concentrations of adiponectin and HDL-cholesterol in the SCT group. There was no association between the markers studied and MS among the groups, however, it is expected, as a pioneering study, that the results obtained may elucidate future investigations.

## Declarations

### Author contribution statement

Jacqueline da Costa Escobar Piccoli: Conceived and designed the experiments; Analyzed and interpreted the data; Contributed reagents, materials, analysis tools or data; Wrote the paper.

Jamila Benvegnú Bruno: Conceived and designed the experiments; Performed the experiments; Analyzed and interpreted the data; Wrote the paper.

Emanuelle Schneider Dal Ponte: Conceived and designed the experiments; Performed the experiments.

Vanessa Retamoso: Performed the experiments.

Patrícia Maurer: Performed the experiments; Analyzed and interpreted the data.

Lyana Feijoó Berro: Performed the experiments.

Vanusa Manfredini: Conceived and designed the experiments; Analyzed and interpreted the data; Contributed reagents, materials, analysis tools or data.

### Funding statement

This study was financed in part by the 10.13039/501100002322Coordenação de Aperfeiçoamento de Pessoal de Nível Superior – Brasil (CAPES) – Finance Code 001.

### Data availability statement

Data will be made available on request.

### Declaration of interests statement

The authors declare no conflict of interest.

### Additional information

No additional information is available for this paper.
